# Implantable Cardiac Monitoring for Atrial Fibrillation Detection in Patients with Stroke: A Systematic Review and Meta-Analysis

**DOI:** 10.3390/neurosci7040083

**Published:** 2026-07-19

**Authors:** Nibras M. Alkhamis, Hussain A. Almohammed, Tanveer N. Khan, Jory H. Alzahrani, Lama A. Alsalboud, Randah T. Alzahrani, Hamad A. Alseni, Amara M. Mufti, Lujeen H. Alghourab, Thekra F. Abuhaimed, Manar M. Alshabaan, Maha E. Alsubaie, Manar A. Jamlalail, Saud A. Alnaaim

**Affiliations:** 1 College of Medicine, King Faisal University, Al Ahsa 31982, Saudi Arabia; nbrasalkhmiss@gmail.com (N.M.A.); sub.maha8@gmail.com (M.E.A.); 2 General Medicine Program, Batterjee Medical College, Jeddah 21442, Saudi Arabia; 3 College of Medicine, King Saud bin Abdulaziz University for Health Sciences, Jeddah 21423, Saudi Arabia; joryzahrani15@gmail.com; 4 College of Pharmacy, Princess Nourah bint Abdulrahman University, Riyadh 11671, Saudi Arabia; lamaalsalboud@gmail.com; 5 College of Medicine, King Abdulaziz University, Jeddah 23452, Saudi Arabia; randahzzz123@gmail.com (R.T.A.); hamadmoutaz@gmail.com (H.A.A.); thekraabuhaimed1@gmail.com (T.F.A.);; 6 College of Medicine, Fakeeh College for Medical Sciences, Jeddah 23323, Saudi Arabia; amaramufti.2004.7@gmail.com; 7 Dammam Medical Complex, Eastern Health Cluster, Dammam 32253, Saudi Arabia; 8 College of Medicine & Surgery, Imam Abdulrahman Bin Faisal University, Dammam 31441, Saudi Arabia; 9 Clinical Neurosciences Department, College of Medicine, King Faisal University, Al Ahsa 31982, Saudi Arabia

**Keywords:** ischemic stroke, transient ischemic attack, implantable cardiac monitor, atrial fibrillation, oral anticoagulation

## Abstract

**Background:** Atrial fibrillation (AF) is a common but frequently undiagnosed cause of ischemic stroke, particularly among patients with cryptogenic stroke and embolic stroke of undetermined source (ESUS). Implantable cardiac monitors (ICMs) enable prolonged continuous rhythm monitoring and may improve AF detection following ischemic stroke or transient ischemic attack (TIA). This systematic review and meta-analysis aimed to evaluate the diagnostic yield, clinical impact, and safety of prolonged ICM monitoring in patients with ischemic stroke or TIA. **Methods:** This systematic review and meta-analysis was conducted in accordance with the PRISMA 2020 guidelines and registered with PROSPERO (CRD42024573913). PubMed, Google Scholar, and the Cochrane Library were systematically searched. Randomized controlled trials and observational studies evaluating the use of ICMs after ischemic stroke or TIA were included. Randomized evidence was synthesized narratively, whereas single-arm random-effects meta-analyses of observational studies were performed to estimate pooled proportions for AF detection, oral anticoagulation initiation, recurrent ischemic stroke or TIA, and device-related adverse events. **Results:** Twelve completed studies involving 4563 participants met the inclusion criteria, including two randomized controlled trials and ten observational studies. One additional ongoing randomized controlled trial (Find-AF 2) involving a planned enrollment of 5200 participants was identified and is described narratively. Across the observational studies, the pooled AF detection rate during prolonged ICM monitoring was 25.9% (95% CI, 18.9–33.5%), although substantial heterogeneity was observed (I^2^ = 95%). Oral anticoagulation was initiated in 94.2% (95% CI, 79.4–100.0%) of patients diagnosed with AF. Device-related complications were uncommon, with a pooled incidence of 3.7% (95% CI, 2.0–6.0%; I^2^ = 0%), while the pooled rate of recurrent ischemic stroke or TIA during follow-up was 6.2% (95% CI, 3.9–9.2%). Narrative synthesis of the randomized evidence demonstrated that ICMs significantly increased AF detection compared with conventional monitoring but did not demonstrate a significant reduction in recurrent stroke during the available follow-up period. **Conclusions:** Prolonged implantable cardiac monitoring identifies AF in approximately one-quarter of patients following ischemic stroke or TIA and frequently leads to the initiation of oral anticoagulation, with a favorable safety profile. Although ICMs substantially improve AF detection, current evidence remains insufficient to confirm that increased detection translates into a reduction in recurrent stroke. Large, adequately powered randomized controlled trials are needed to determine the long-term clinical benefits of ICM-guided management and to define the optimal monitoring strategy for patients following ischemic stroke.

## 1. Introduction

Stroke is a major global cause of mortality and disability, with ischemic stroke accounting for the majority of cases worldwide [1,2]. Up to one-third of ischemic strokes are attributable to atrial fibrillation (AF) [3]. AF promotes blood stasis, resulting in a prothrombotic state that increases the risk of thromboembolic events [4]. However, the burden of AF is often underestimated because of undetected paroxysmal AF episodes [5]. Patients with ischemic stroke and underlying AF have an approximately twofold higher risk of recurrent ischemic stroke than those without AF [6].

Furthermore, ischemic strokes associated with AF are generally more severe and are associated with higher rates of disability and mortality, worse functional outcomes, and lower rates of discharge to home [7,8]. Consequently, timely detection of AF is essential for preventing recurrent ischemic stroke and initiating appropriate oral anticoagulation therapy. Diagnosing AF is therefore a key objective in the management of ischemic stroke and transient ischemic attack (TIA). However, paroxysmal AF frequently remains undiagnosed because of its short duration, asymptomatic presentation, and intermittent recurrence [9].

Current guidelines recommend at least 24 h of cardiac monitoring following an ischemic stroke [10]. However, accumulating evidence suggests that prolonged monitoring, particularly with implantable cardiac monitors (ICMs), substantially improves AF detection. In 2022, Tsivgoulis et al. reported that extended cardiac monitoring was associated with a lower risk of first or recurrent ischemic stroke, higher AF detection rates, and increased initiation of oral anticoagulation among patients with cardiovascular risk factors, including those with a history of ischemic stroke [7]. Current recommendations also support prolonged cardiac monitoring for up to six months in selected patients with cryptogenic stroke [11,12]. Furthermore, a 2023 study demonstrated that ICM use was associated with a 24% reduction in ischemic stroke incidence compared with conventional external cardiac monitoring in patients with a history of stroke or other stroke risk factors [5]. These studies also demonstrated higher AF detection rates and greater initiation of oral anticoagulation among patients undergoing ICM monitoring [4,5]. Nevertheless, despite improving AF detection and anticoagulation use, ICMs have not consistently been associated with significant reductions in recurrent stroke or all-cause mortality [4].

Despite these promising findings, important clinical questions remain regarding the optimal selection of patients for ICM implantation, the appropriate duration of monitoring, and which patients with device-detected AF derive the greatest benefit from anticoagulation for secondary stroke prevention. Therefore, this systematic review and meta-analysis aimed to evaluate the efficacy and safety of ICM strategies for detecting AF and their impact on recurrent stroke and clinical outcomes in patients with ischemic stroke or TIA.

## 2. Methods

### 2.1. Protocol Approval and Registration

This systematic review and meta-analysis was conducted and reported in accordance with the PRISMA 2020 guidelines [13]. The study protocol was prospectively registered with PROSPERO (registration number: CRD42024573913). Because this study involved the synthesis of previously published data, institutional review board approval and informed patient consent were not required.

### 2.2. Literature Search Strategy

A comprehensive literature search was conducted in PubMed, the Cochrane Library, and Google Scholar from database inception through 26 July 2025. Two independent reviewers performed the literature search using a predefined search strategy. To ensure a comprehensive and unbiased literature search, PubMed and the Cochrane Library were used as the primary electronic databases for identifying eligible studies. Google Scholar was searched as a supplementary source of the gray literature using multiple combinations of the predefined search terms. Searches were conducted independently using different keyword combinations to maximize retrieval of potentially relevant studies. All retrieved records were exported and combined, and duplicate citations were removed before screening. To improve reproducibility, screening was limited to the first 230 pages of Google Scholar search results (approximately 2300 records, with 10 results displayed per page) sorted by relevance. Searches were performed using predefined keyword combinations, and all retrieved citations were exported to Rayyan for duplicate removal and screening. Additionally, the reference lists of all included studies and relevant reviews were manually searched to identify any further eligible publications. Reference lists of the included studies and relevant reviews were also manually screened to identify any additional eligible publications. The following search terms were used: (“Cardiac Monitors” OR “Insertable Cardiac Monitors” OR “Implantable Cardiac Monitors” OR “ICM” OR “ILR” OR “Insertable Loop Recorders” OR “Implantable Loop Recorders” OR “External Loop Recorders” OR “Holter Monitors” OR “Prolonged Cardiac Monitoring”) AND (“Ischemic Stroke” OR “Stroke” OR “TIA” OR “Cryptogenic Stroke” OR “ESUS” OR “Cerebral Ischemia”) AND (“AF” OR “Atrial Fibrillation”).

### 2.3. Eligibility Criteria

Eligible studies included randomized controlled trials and prospective or retrospective observational cohort studies evaluating implantable cardiac monitoring in adults (≥18 years) with ischemic stroke or TIA, including cryptogenic stroke and embolic stroke of undetermined source (ESUS). Conventional cardiac monitoring strategies, including serial electrocardiography, Holter monitoring, and external loop recorders, were considered comparator strategies where applicable. Studies were excluded if they did not evaluate implantable cardiac monitoring after ischemic stroke or TIA, did not report outcomes relevant to this review, or were case reports, case series, narrative reviews, conference abstracts without sufficient data, editorials, letters, animal studies, or previous systematic reviews and meta-analyses.

### 2.4. Study Selection

Two independent reviewers screened all retrieved records by title and abstract using the Rayyan web application according to the predefined eligibility criteria [14]. Full-text articles considered potentially eligible were subsequently assessed independently by three reviewers. Any disagreements during either the title and abstract screening or the full-text review were resolved through discussion with a third and fourth reviewer until consensus was reached. During title and abstract screening, Rayyan’s automation features were used to assist in identifying duplicate and clearly irrelevant records. All records flagged by automation were subsequently reviewed by the investigators before exclusion, ensuring that no potentially eligible studies were removed without manual verification.

### 2.5. Outcomes and Data Synthesis

Data were synthesized using both qualitative and quantitative approaches. Characteristics of the included studies were summarized descriptively, including study design, patient population, sample size, participant demographics, monitoring duration, AF detection criteria, comparator strategies, follow-up duration, and reported clinical outcomes.

Where at least three studies reported comparable outcomes, quantitative synthesis was performed using single-arm meta-analysis of proportions. The primary pooled outcomes included AF detection rate during ICM follow-up, device-related adverse events, recurrent ischemic stroke or TIA during follow-up, and oral anticoagulation initiation following AF detection. AF detection thresholds were used across the included studies. These thresholds included episodes lasting ≥30 s and ≥2 min, device-programmed criteria, CRYSTAL-AF criteria, and longer AF burden definitions such as ≥6 min or ≥5.5 h.

Because several studies reported proportions close to 0% or 100%, pooled prevalence estimates were calculated using the Freeman–Tukey double-arcsine transformation to stabilize variances before pooling. Random-effects models were applied using the DerSimonian–Laird estimator to account for anticipated clinical and methodological heterogeneity among studies, including differences in patient populations, AF detection thresholds, monitoring duration, and study design. Pooled estimates are presented as proportions with corresponding 95% confidence intervals (95% CIs).

Statistical heterogeneity was evaluated using Cochran’s Q test and quantified with the I^2^ statistic. Heterogeneity was interpreted according to conventional thresholds, with I^2^ values of approximately 25%, 50%, and 75% representing low, moderate, and high heterogeneity, respectively. Statistical significance for heterogeneity was defined as *p* < 0.10 for Cochran’s Q test.

Only studies reporting sufficient numerical data for each outcome were included in the corresponding meta-analysis. Studies that reported outcomes qualitatively without extractable numerical data were included in the narrative synthesis but were excluded from quantitative pooling. Statistical analyses were performed using R statistical software (version 4.4.1; R Foundation for Statistical Computing, Vienna, Austria). Meta-analyses were conducted using the meta package (version 8.1-0) and the metafor package (version 4.8-0).

### 2.6. Risk of Bias Assessment

The methodological quality of the included studies was independently assessed by two reviewers. Randomized controlled trials were evaluated using the Cochrane Risk of Bias 2 (RoB 2) tool [15], which assesses bias arising from the randomization process, deviations from intended interventions, missing outcome data, outcome measurement, and selective reporting. Each domain was classified as low risk of bias, some concerns, or high risk of bias, leading to an overall study-level judgment.

Observational cohort studies were assessed using the Newcastle–Ottawa Scale (NOS) [16], which evaluates methodological quality across three domains: participant selection, comparability of study groups, and ascertainment of outcomes. Studies were awarded a maximum of nine stars and categorized as high quality (7–9 stars), moderate quality (5–6 stars), or low quality (<5 stars). Disagreements between reviewers were resolved through discussion until consensus was achieved.

### 2.7. Certainty of Evidence Assessment

The certainty of evidence for each pooled outcome was assessed using the Grading of Recommendations Assessment, Development and Evaluation (GRADE) framework [17]. Certainty was evaluated separately for AF detection, device-related adverse events, recurrent ischemic stroke or TIA, and oral anticoagulation initiation following AF detection.

Observational evidence was initially rated as low certainty, whereas randomized evidence was initially rated as high certainty, with subsequent downgrading based on risk of bias, inconsistency, indirectness, imprecision, and publication bias. Evidence was upgraded when appropriate according to GRADE recommendations. Final certainty ratings were classified as high, moderate, low, or very low.

## 3. Results

**Figure 1:**
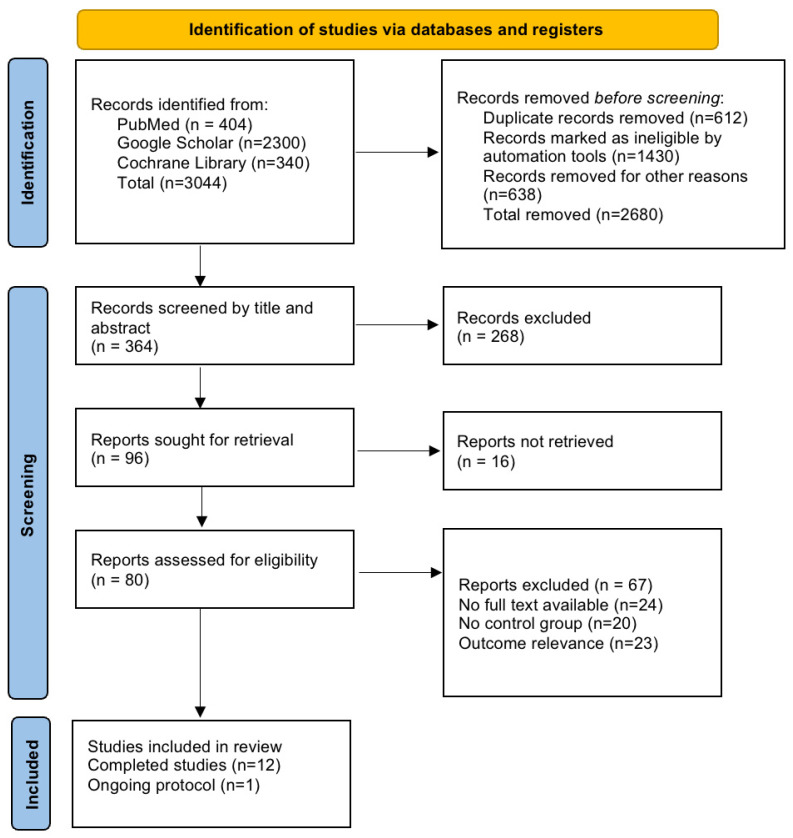
PRISMA 2020 flow diagram illustrating the identification, screening, eligibility assessment, and inclusion of completed studies in the evidence synthesis, with the ongoing Find-AF 2 trial reported narratively only.

The literature search identified a total of 3044 records from three electronic databases: PubMed (n = 404), Google Scholar (n = 2300), and the Cochrane Library (n = 340). Following the removal of duplicate records (n = 612), records identified as clearly irrelevant during Rayyan-assisted screening (n = 1430), and records excluded for other reasons (n = 638), 364 unique records remained for title and abstract screening. Of these, 268 records were excluded based on the eligibility criteria, leaving 96 reports for full-text retrieval. Sixteen reports could not be retrieved, leaving 80 reports for full-text assessment. Following full-text review, 67 studies were excluded because they did not meet the predefined eligibility criteria, including inappropriate study population, intervention, study design, or outcome reporting. Consequently, 13 studies fulfilled the predefined eligibility criteria. Twelve completed studies (two randomized controlled trials and ten observational studies) were included in the evidence synthesis. One additional ongoing randomized controlled trial (Find-AF 2) was identified and is described narratively because completed clinical outcome data were not yet available. Accordingly, Find-AF 2 was not included in the quantitative synthesis, risk-of-bias assessment, or GRADE evaluation [18,19,20,21,22,23,24,25,26,27,28,29,30]. The detailed study selection process is illustrated in Figure 1.

### 3.1. Characteristics of Included Studies

Two completed randomized controlled trials involving 912 participants were included, evaluating implantable cardiac monitoring following ischemic stroke, cryptogenic stroke, or transient ischemic attack (Table 1). The completed randomized controlled trials were conducted in the United States and multinational settings, with sample sizes of 441 and 471 participants, respectively. Eligible participants were generally older adults (≥40–60 years), and males comprised approximately 62–64% of the reported study populations. Hypertension and diabetes mellitus were the most prevalent baseline comorbidities, while heart failure, vascular disease, smoking, and patent foramen ovale were also frequently reported. Monitoring duration ranged from 12 to 36 months. Two trials defined AF as episodes lasting at least 30 s, whereas the AF definition was not specified in the Find-AF 2 protocol [20]. Across both completed randomized studies, ICM was compared with conventional cardiac rhythm monitoring strategies, including electrocardiography, Holter monitoring, telemetry, and event recorders.

One additional randomized controlled trial (Find-AF 2) was identified during the literature search. This multicenter German trial plans to enroll approximately 5200 participants with follow-up extending to six years. As the study is ongoing and clinical outcome data have not yet been reported, it was described narratively only and was not included in the evidence synthesis, risk-of-bias assessment, or GRADE evaluation.

Ten observational studies comprising 3651 patients were included, encompassing prospective and retrospective cohort studies, prospective observational studies, multicentre registries, and registry-based analyses (Table 2). These studies were conducted across Europe, the United Kingdom, the United States, and multinational settings, with sample sizes varying substantially from 51 to 1262 participants. The study populations predominantly consisted of patients with cryptogenic stroke, ESUS, or stroke/TIA. Participants had a mean or median age ranging from approximately 52 to 70 years, and males represented 50–67% of the study populations. Reported CHA_2_DS_2_-VASc scores generally ranged between 3 and 5, indicating a moderate-to-high baseline risk of thromboembolism. Continuous rhythm monitoring was performed for periods extending from approximately seven months to three years. Most studies defined AF as episodes lasting at least two minutes, although alternative thresholds, including ≥30 s, device-programmed criteria, CRYSTAL-AF criteria, and multiple detection thresholds, were also used. Follow-up durations were largely consistent with monitoring periods, enabling long-term evaluation of AF detection and associated clinical outcomes.

### 3.2. Clinical Outcomes of Implantable Cardiac Monitoring

The clinical outcomes of the included studies are summarized in Table 3. Across the randomized controlled trials and observational studies, prolonged cardiac rhythm monitoring using implantable cardiac monitors consistently improved the detection of AF following ischemic stroke or transient ischemic attack. Reported AF detection rates varied considerably, ranging from 5.5% to 48.6%, largely reflecting differences in patient populations, AF detection thresholds, monitoring duration, and study design. In general, studies with longer follow-up periods demonstrated progressively higher cumulative AF detection rates, highlighting the incremental diagnostic yield of extended continuous monitoring. The time to first AF detection also varied substantially across studies, with median or mean detection times ranging from 48 days to approximately 8 months after device implantation. Several studies reported that a considerable proportion of AF episodes were identified beyond the initial six months of monitoring, emphasizing the limitations of short-term external cardiac monitoring for detecting paroxysmal AF. Where reported, recurrent ischemic stroke or transient ischemic attack occurred infrequently during follow-up, with recurrence rates ranging from 3.0% to 9.9%. Identification of AF frequently resulted in a change in secondary stroke prevention strategy, with most studies reporting initiation of oral anticoagulation in the majority of or all patients diagnosed with AF. Among studies providing treatment data, anticoagulation initiation ranged from 60.5% to 100% following AF detection. Overall, implantable cardiac monitors demonstrated an excellent safety profile. Device-related complications were uncommon and predominantly minor, including implant-site pain, hematoma, localized infection, or battery depletion. Major complications requiring device explantation were rare, and no study reported unexpected safety concerns attributable to prolonged implantable cardiac monitoring.

Although the present quantitative synthesis focused on observational studies, evidence from randomized controlled trials has previously been systematically evaluated. Tan et al. (2021) conducted a meta-analysis of two randomized controlled trials comparing ICMs with conventional cardiac rhythm monitoring in patients with ischemic stroke or transient ischemic attack [31]. They reported that prolonged ICM monitoring significantly increased the detection of AF at both 6- and 12-month follow-up compared with conventional monitoring, confirming the superior diagnostic yield of continuous implantable monitoring. However, no significant difference was observed between groups in the time to first AF detection or in the incidence of recurrent ischemic stroke or transient ischemic attack during the first 12 months of follow-up. The review also demonstrated greater use of oral anticoagulation among patients monitored with ICMs following AF detection, reflecting the clinical impact of identifying previously undiagnosed AF. With respect to safety, device implantation was associated with a slightly higher incidence of procedure-related adverse events, including implant-site pain, infection, inflammation, and hematoma; however, these complications were uncommon and generally minor. Overall, Tan et al. [31] concluded that ICMs are superior to conventional monitoring for post-stroke AF detection while maintaining an acceptable safety profile, although additional large, high-quality randomized trials are required to determine whether the increased detection of AF ultimately translates into improved long-term clinical outcomes.

### 3.3. AF Detection Rate on ICM Monitoring

**Figure 2:**
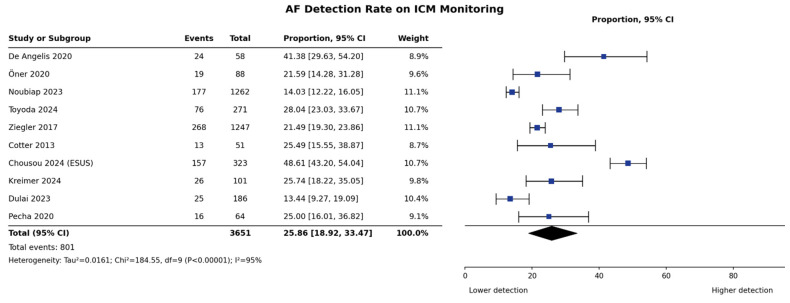
Random-effects single-arm meta-analysis of atrial fibrillation detection during implantable cardiac monitor follow-up among observational cohort studies of patients with ischemic stroke, cryptogenic stroke, ESUS, or transient ischemic attack [21,22,23,24,25,26,27,28,29,30].

Ten single-arm cohorts (n = 3651 patients total) reported the proportion of cryptogenic stroke, ESUS, or TIA patients in whom atrial fibrillation was detected during ICM follow-up. Individual study estimates ranged widely, from 13.4% (Dulai 2023) [26] to 48.6% (Chousou 2024, ESUS subgroup) [29], and the pooled random-effects estimate was 25.9% (95% CI 18.9–33.5%). Heterogeneity was very high (I^2^ = 95%, χ^2^ = 184.55, df = 9, *p* < 0.0001), driven by genuine differences in monitoring duration, AF-detection thresholds, and the underlying index event across studies, so the pooled estimate should be interpreted as a descriptive summary across heterogeneous clinical settings rather than a single universally applicable estimate. The observed heterogeneity most likely reflects differences in stroke subtype, monitoring duration, atrial fibrillation detection thresholds, and study design (Figure 2).

#### 3.3.1. Device-Related Adverse Events 

**Figure 3:**
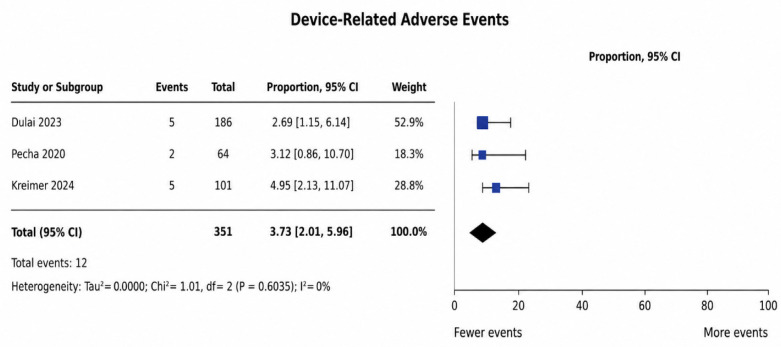
Random-effects single-arm meta-analysis of device-related adverse events associated with implantable cardiac monitoring among observational cohort studies [25,26,30].

Three cohorts (n = 351 patients) reported raw counts of ICM-related complications such as device infection and hematoma. Estimates were tightly clustered, from 2.7% (Dulai 2023) [26] to 5.0% (Kreimer 2024) [30], giving a pooled random-effects complication rate of 3.7% (95% CI 2.0–6.0%) with no detected heterogeneity (I^2^ = 0%, χ^2^ = 1.01, df = 2, *p* = 0.60). With only 3 small studies contributing, this result should be treated as hypothesis-generating rather than a definitive safety estimate (Figure 3).

#### 3.3.2. Recurrent Ischemic Stroke or TIA During ICM Follow-Up 

**Figure 4:**
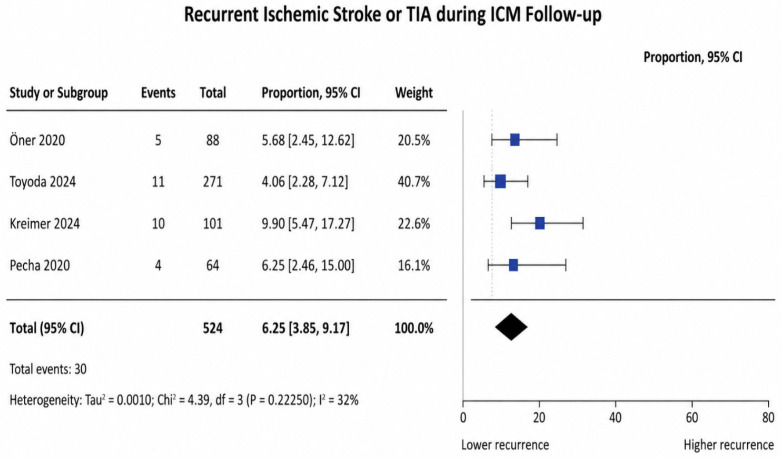
Random-effects single-arm meta-analysis of recurrent ischemic stroke or transient ischemic attack during follow-up among observational cohort studies of patients undergoing implantable cardiac monitoring [24,25,28,30].

Four cohorts (n = 524 patients) reported a raw count of recurrent ischemic stroke or TIA during ICM follow-up. Individual estimates were fairly consistent, ranging from 4.1% (Toyoda 2024) [28] to 9.9% (Kreimer 2024) [30], with a pooled random-effects recurrence rate of 6.2% (95% CI 3.9–9.2%). Heterogeneity was low-to-moderate (I^2^ = 32%, χ^2^ = 4.39, df = 3, *p* = 0.22), indicating this estimate is considerably more internally consistent than the AF-detection outcome above, though it still rests on a small number of studies (Figure 4).

### 3.4. Oral Anticoagulation Initiation Following AF Detection on ICM Monitoring

**Figure 5:**
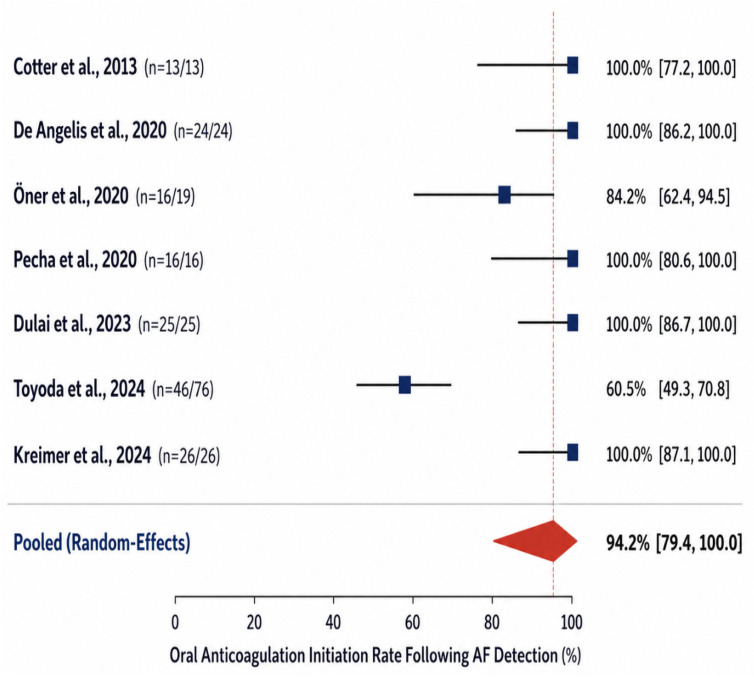
Random-effects single-arm meta-analysis of oral anticoagulation initiation following atrial fibrillation detection via implantable cardiac monitoring among observational cohort studies [21,23,24,25,26,28,30].

Study-level anticoagulation initiation rates ranged from 60.5% (Toyoda 2024) [28] to 100% (Cotter 2013, De Angelis 2020, Pecha 2020, Dulai 2023, Kreimer 2024) [21,23,25,26,30] (Figure 5). Proportions were pooled using a random-effects model with the Freeman–Tukey double-arcsine transformation and DerSimonian–Laird tau^2^ estimation, appropriate for binomial proportions including those close to 100%. The pooled anticoagulation initiation rate was 94.2% (95% CI 79.4–100.0%), with substantial heterogeneity (I^2^ = 89.9%, χ^2^ = 59.19, df = 6, *p* < 0.0001) driven primarily by the comparatively lower initiation rate observed in Toyoda 2024 [28]. Excluding this study, the remaining six cohorts were homogeneous, each reporting anticoagulation in 84–100% of AF-positive patients. These findings indicate that, across the included studies, detection of AF via ICM monitoring was followed by initiation of oral anticoagulation in the large majority of patients, supporting the clinical actionability of prolonged cardiac rhythm monitoring after ischemic stroke, cryptogenic stroke, or TIA.

### 3.5. Risk of Bias Assessment

**Figure 6:**
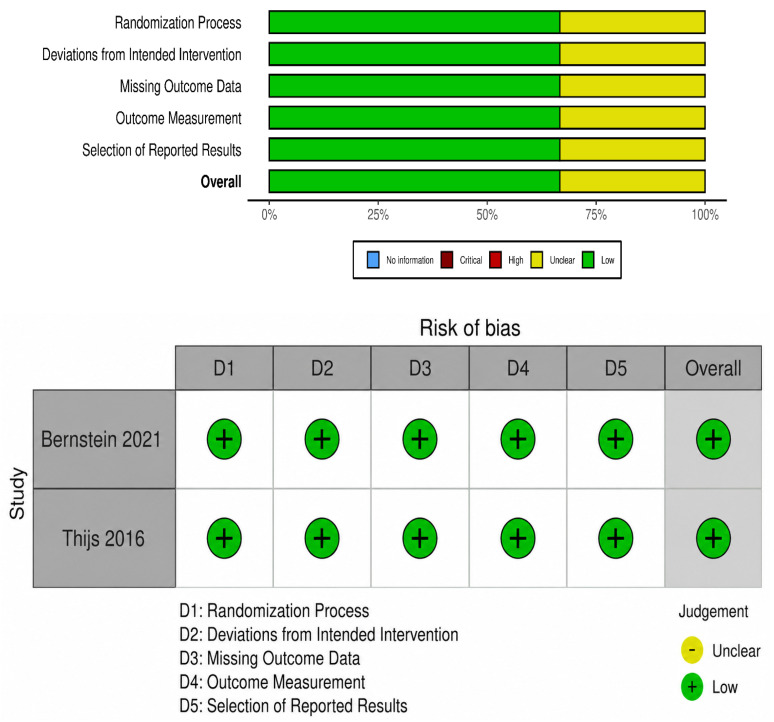
Risk of bias assessment of the completed randomized controlled trials using the Cochrane Risk of Bias 2 (RoB 2) tool. Green circles indicate low risk of bias across all assessed domains. The ongoing Find-AF 2 trial was not assessed because clinical outcome data were not available [18,19].

Among the two randomized controlled trials, two studies were judged to have an overall low risk of bias, demonstrating adequate randomization procedures, minimal missing outcome data, objective outcome assessment, and a low likelihood of selective reporting. The ongoing Find-AF 2 trial was not assessed using RoB 2 because no outcome data were available for evaluation. Overall, the randomized evidence was considered to be of good methodological quality (Figure 6).

**Figure 7:**
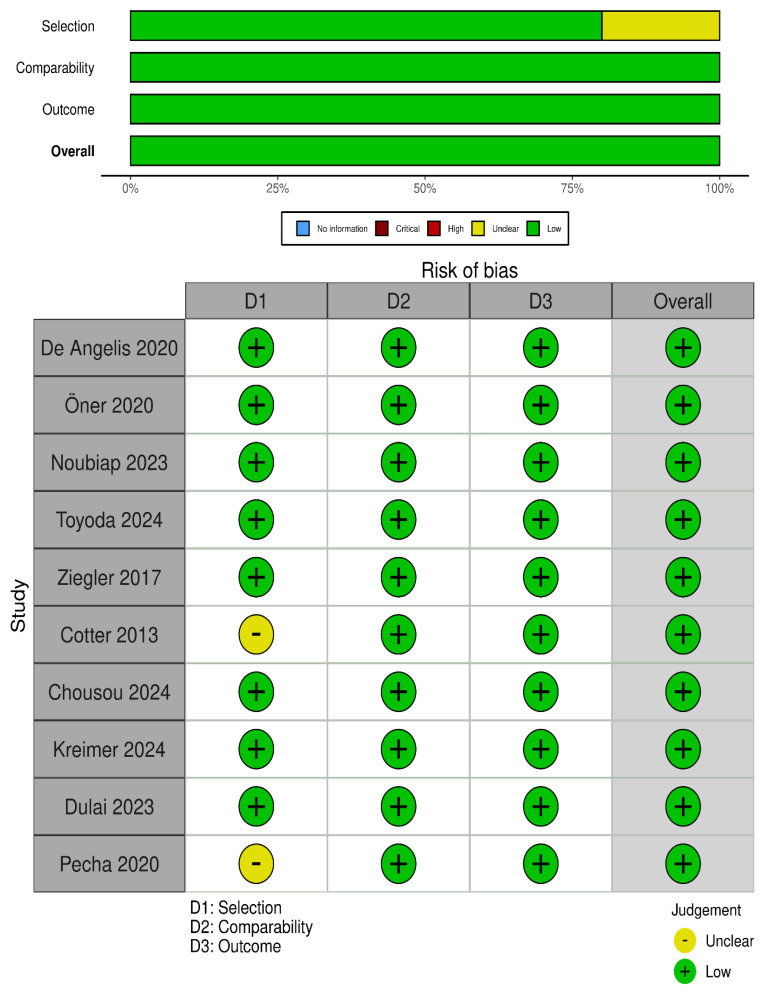
Methodological quality assessment of the included observational cohort studies using the Newcastle–Ottawa Scale (NOS). Higher scores indicate better methodological quality and lower risk of bias [21,22,23,24,25,26,27,28,29,30].

The observational studies demonstrated generally high methodological quality (Figure 7). NOS scores ranged from 7 to 9 stars, with the majority of studies achieving 8 or 9 stars, indicating a low risk of bias. Most studies employed representative patient populations, clearly defined inclusion criteria, adequate follow-up durations, and objective methods for atrial fibrillation detection using implantable cardiac monitors. Minor limitations were primarily related to the retrospective design of several studies, limited adjustment for potential confounders, and the single-center nature of some cohorts. Nevertheless, these limitations were not considered sufficient to substantially compromise the overall validity of the findings.

### 3.6. Certainty of Evidence (GRADE)

The certainty of evidence varied across the evaluated outcomes depending on methodological quality, consistency of findings, and precision of the pooled estimates (Table 4) [17]. The ongoing Find-AF 2 trial was not incorporated into the certainty assessment because completed clinical outcome data were unavailable.

### 3.7. AF Detection Rate Following ICM Monitoring

The certainty of evidence for AF detection was rated as low. Although all included observational studies consistently demonstrated that prolonged implantable cardiac monitoring substantially increased AF detection, the evidence originated exclusively from observational cohorts. Furthermore, the pooled analysis demonstrated considerable statistical heterogeneity (I^2^ = 95%), largely attributable to differences in patient populations, monitoring duration, AF detection thresholds, and study protocols. No serious concerns regarding indirectness were identified, while publication bias could not be formally assessed because of the limited number of studies.

### 3.8. Device-Related Adverse Events

The certainty of evidence for device-related complications was judged to be low. The pooled complication rate was low (3.7%) with no observed heterogeneity (I^2^ = 0%), suggesting highly consistent findings across studies. However, only three relatively small observational cohorts contributed data, resulting in imprecision due to limited sample size and few reported adverse events. Consequently, the overall certainty remained low.

### 3.9. Recurrent Ischemic Stroke or TIA

The certainty of evidence for recurrent ischemic stroke or TIA during ICM follow-up was rated as low. Four observational studies contributed to this outcome, demonstrating a relatively consistent pooled recurrence rate (6.2%) with only low-to-moderate heterogeneity (I^2^ = 32%). Nevertheless, the small number of studies, limited number of events, and observational design resulted in downgrading for imprecision and study limitations.

### 3.10. Oral Anticoagulation Initiation Following AF Detection

The certainty of evidence for oral anticoagulation initiation following AF detection was rated as very low. Although most studies reported anticoagulation initiation in nearly all patients diagnosed with AF, substantial heterogeneity (I^2^ = 89.9%) was observed, primarily driven by one study reporting a lower treatment initiation rate. Additionally, the evidence originated solely from observational cohorts with relatively small sample sizes, leading to downgrading for risk of bias, inconsistency, and imprecision.

Overall, the certainty of evidence supporting the clinical benefits of implantable cardiac monitoring ranged from low to very low, reflecting the predominance of observational evidence despite generally consistent findings across studies. Higher-quality randomized evidence evaluating clinically important outcomes beyond AF detection, including recurrent stroke prevention and treatment implementation, is warranted.

## 4. Discussion

This systematic review and meta-analysis evaluated the diagnostic yield, clinical impact, and safety of ICM following ischemic stroke or TIA, including patients with cryptogenic stroke and ESUS. Across ten observational studies, approximately one-quarter of patients undergoing prolonged ICM monitoring were diagnosed with AF, highlighting the substantial diagnostic yield of continuous rhythm monitoring in this population. Moreover, AF detection led to the initiation of oral anticoagulation in the vast majority of affected patients, whereas device-related complications were uncommon. Although recurrent ischemic stroke or TIA occurred infrequently during follow-up, current evidence remains insufficient to determine whether improved AF detection ultimately translates into reduced recurrent stroke or other long-term clinical benefits.

Our findings are consistent with the randomized evidence summarized by Tan et al. (2021) [31], who demonstrated that ICM significantly increases AF detection compared with conventional cardiac monitoring following ischemic stroke or TIA, although no significant reduction in recurrent stroke was observed during the available follow-up period. These findings support the diagnostic utility of prolonged ICM monitoring while highlighting the need for further randomized evidence evaluating patient-important outcomes [31].

### 4.1. AF Detection and the Value of Prolonged Monitoring

Our pooled analysis demonstrated an overall AF detection rate of 25.9%, although substantial between-study heterogeneity was observed. This variability is likely attributable to important clinical differences among the included studies, including monitoring duration, AF detection thresholds, patient populations, and index cerebrovascular events. For example, studies including patients with ESUS generally reported higher AF detection rates than cohorts restricted to cryptogenic stroke, while definitions of AF ranged from episodes lasting ≥30 s to ≥2 min or device-specific detection algorithms.

Despite this heterogeneity, a consistent pattern emerged across studies: AF detection increased progressively with longer monitoring durations. Several cohorts demonstrated that a considerable proportion of AF episodes were identified beyond the first six months following device implantation, emphasizing the limitations of conventional short-term monitoring for detecting intermittent or asymptomatic paroxysmal AF.

These findings are consistent with the previous literature. Tsivgoulis et al. [7] reported significantly higher AF detection among patients undergoing ICM compared with conventional monitoring. Furthermore, randomized evidence synthesized by Tan et al. [31] confirmed significantly greater AF detection with ICM than with conventional monitoring strategies, reinforcing the role of prolonged continuous rhythm surveillance after ischemic stroke.

Although prolonged monitoring appears advantageous, the optimal duration of ICM monitoring remains uncertain. The included studies employed monitoring periods ranging from 12 months to more than three years, and no study was specifically designed to determine the ideal monitoring duration. Nevertheless, the continued increase in cumulative AF detection throughout follow-up suggests that extended monitoring provides incremental diagnostic benefit, particularly in patients at high risk of occult AF.

### 4.2. Clinical Actionability: Oral Anticoagulation Following AF Detection

An important finding of this review is that AF detection through prolonged ICM monitoring was highly clinically actionable. Across seven observational studies, the pooled rate of oral anticoagulation initiation following AF diagnosis was 94.2%, indicating that identification of previously undiagnosed AF frequently resulted in modification of secondary stroke prevention strategies.

Although substantial heterogeneity was observed, this was largely driven by the comparatively lower anticoagulation initiation rate reported by Toyoda et al. [28]. Several factors may explain this difference, including variation in atrial fibrillation burden thresholds used to initiate anticoagulation, differences in local prescribing practices, physician assessment of bleeding risk, and ongoing uncertainty regarding the clinical significance of brief subclinical atrial fibrillation episodes detected by prolonged monitoring. Consequently, anticoagulation decisions remain individualized despite increased atrial fibrillation detection.

These findings align with randomized evidence summarized by Tan et al. [31], who similarly observed greater anticoagulant use among patients undergoing ICM compared with conventional monitoring following AF detection. Collectively, these observations suggest that prolonged rhythm monitoring has substantial clinical utility by identifying patients eligible for evidence-based anticoagulation therapy. An additional large randomized trial, Find-AF 2, has published its study protocol and baseline characteristics but has not yet reported clinical outcomes. Once available, the results of this trial may provide important evidence regarding whether prolonged implantable cardiac monitoring improves clinically meaningful outcomes beyond increased atrial fibrillation detection, including recurrent stroke prevention and long-term anticoagulation strategies.

However, the increasing sensitivity of prolonged monitoring also raises important clinical questions regarding the AF burden required to justify anticoagulation. As ICMs detect progressively shorter and less frequent AF episodes, further research is required to determine the threshold at which anticoagulation provides net clinical benefit while minimizing bleeding risk.

### 4.3. Safety of Implantable Cardiac Monitoring

Our pooled analysis demonstrated a low device-related complication rate of 3.7%, with no evidence of statistical heterogeneity. Reported complications were predominantly minor and included implant-site pain, localized infection, hematoma, battery depletion, and occasional device explantation. Serious device-related complications were uncommon across all included studies.

These findings are consistent with previous studies evaluating ICM safety. Buck et al. and Quartieri et al. similarly reported low complication rates, with the vast majority of patients completing long-term monitoring without significant adverse events [32,33]. Collectively, the available evidence supports the favorable safety profile of implantable cardiac monitors, particularly when balanced against their high diagnostic yield.

### 4.4. Recurrent Stroke and Remaining Clinical Uncertainties

Although recurrent ischemic stroke or TIA occurred relatively infrequently during ICM follow-up, with a pooled recurrence rate of 6.2%, the available evidence does not permit conclusions regarding the effectiveness of ICMs in reducing recurrent cerebrovascular events.

Importantly, the observational studies included in this review were not designed to compare recurrence between monitoring strategies. Likewise, randomized evidence summarized by Tan et al. [31] found no significant reduction in recurrent stroke despite substantially greater AF detection with ICM. Consequently, while earlier AF detection facilitates anticoagulation initiation, whether this ultimately translates into improved long-term clinical outcomes remains uncertain.

Future randomized trials with longer follow-up are therefore required to determine whether ICM-guided management reduces recurrent stroke, systemic embolism, mortality, or functional disability.

### 4.5. Clinical Implications

The findings of this review have important implications for secondary stroke prevention. Approximately one in four patients undergoing prolonged ICM monitoring after ischemic stroke or TIA was diagnosed with AF, and nearly all patients subsequently initiated oral anticoagulation. These findings support the role of prolonged continuous rhythm monitoring in selected patients at increased risk of occult AF, particularly those with cryptogenic stroke or ESUS.

Nevertheless, patient selection remains essential. Older age and other established AF risk factors may identify individuals most likely to benefit from prolonged monitoring while improving cost-effectiveness and resource utilization. Future studies should also compare implantable monitoring with emerging non-invasive technologies, including extended-wear ECG patches and wearable devices, to determine the most effective and economically sustainable monitoring strategy across different healthcare settings.

### 4.6. Strengths and Limitations

This study has several strengths. To our knowledge, it is among the few systematic reviews to comprehensively synthesize both randomized and observational evidence regarding prolonged implantable cardiac monitoring after ischemic stroke while additionally pooling clinically relevant outcomes including AF detection, oral anticoagulation initiation, recurrent stroke, and device-related complications. The methodological quality of the included studies was generally high, and certainty of evidence was evaluated using the GRADE framework.

Several limitations should also be acknowledged. First, all quantitative meta-analyses were derived from observational studies because randomized trials did not provide sufficient extractable data for pooled analyses. Second, considerable heterogeneity was observed for AF detection and anticoagulation initiation, reflecting differences in monitoring duration, AF detection thresholds, study populations, and clinical practice. Third, evidence regarding recurrent stroke and other patient-important outcomes remains limited, with most studies focusing primarily on AF detection rather than clinical endpoints. Fourth, publication bias could not be formally assessed because of the relatively small number of studies contributing to each outcome. Finally, the included studies were predominantly conducted in high-income healthcare systems, which may limit the generalizability of these findings to lower-resource settings.

## 5. Conclusions

Prolonged implantable cardiac monitoring following ischemic stroke, cryptogenic stroke, ESUS, or TIA identifies atrial fibrillation in approximately one-quarter of monitored patients and results in initiation of oral anticoagulation in the vast majority of those diagnosed. Device-related complications are uncommon, supporting the overall safety of long-term implantable monitoring. However, despite its high diagnostic yield and clear impact on clinical management, current evidence remains insufficient to demonstrate that increased AF detection translates into reductions in recurrent stroke or other major clinical outcomes. Large, adequately powered randomized trials with extended follow-up are required to determine whether ICM-guided management improves long-term patient outcomes and to define the optimal monitoring duration and patient populations most likely to benefit.

## Figures and Tables

**Table 1:** Characteristics of Completed Randomized Controlled Trials.

Study	Country	Study Design	Study Population	Sample Size (ICM/Control)	Age (Years)	Male (%)	Index Event	Major Comorbidities	Monitoring Duration	AF Definition	Comparator	Follow-Up
Bernstein et al., 2021 (STROKE-AF) [18]	USA	Randomized Clinical Trial	Ischemic stroke attributed to large- or small-vessel disease	221/250	≥50	62.4	Ischemic stroke	Hypertension, diabetes mellitus, smoking, vascular disease, heart failure	12 months	≥30 s	Conventional cardiac monitoring (ECG, Holter, telemetry, event recorder)	12 months
Thijs et al., 2016 (CRYSTAL-AF) [19]	Multinational	Randomized Controlled Trial	Cryptogenic stroke or TIA	221/220	≥40	64.3	Stroke/TIA	Hypertension, diabetes mellitus, heart failure, patent foramen ovale	12–36 months	≥30 s	Conventional follow-up (Holter monitoring/telemetry)	36 months

Abbreviations: AF, atrial fibrillation; ECG, electrocardiogram; ICM, implantable cardiac monitor; NR, not reported; TIA, transient ischemic attack.

**Table 2:** Characteristics of Included Observational Studies.

Study	Country	Study Design	Population	Sample Size	Age (Years)	Male (%)	CHA_2_DS_2_-VASc	Monitoring Duration	AF Cut-Off	Follow-Up
Cotter et al., 2013 [21]	UK	Prospective cohort	Cryptogenic stroke	51	Median 52 (17–73)	54.9	Median 3	Mean 229 days	≥2 min	Mean 229 days
Ziegler et al., 2017 [22]	USA	Retrospective cohort	Cryptogenic stroke	1247	65.3 ± 13.0	53.0	NR	Up to 24 months	≥2 min	Mean 579 days
De Angelis et al., 2020 [23]	Italy	Prospective observational	Cryptogenic stroke	58	68.1 ± 9.3	67.0	4.4 ± 1.4	Up to 36 months	≥2 min	Mean 906 days
Öner et al., 2020 [24]	Germany	Prospective observational	Stroke/TIA	88	Median 66.5	61.4	Median 4	Median 21.5 months	Device programmed	Median 21.5 months
Pecha et al., 2020 [25]	Germany	Retrospective cohort	Cryptogenic stroke	64	65.4 ± 12.0	50.0	4.7 ± 1.4	Mean 14 months	CRYSTAL-AF criteria	Mean 419 days
Dulai et al., 2023 [26]	UK	Retrospective cohort	Cryptogenic stroke	186	68.7 ± 10.8	63.8	4.3 ± 1.3	Mean 12 months	≥2 min	Mean 363 days
Noubiap et al., 2023 [27]	USA	Multicentre retrospective cohort	Stroke/TIA	1262	69.7 ± 12.2	58.9	NR	Median 26 months	≥2 min	Median 26 months
Toyoda et al., 2024 [28]	International (12 countries)	Prospective registry	Cryptogenic stroke/TIA	271	61.6 ± 14.3	62.7	Median 4	Up to 36 months	≥2 min	Median 20 months
Chousou et al., 2024 [29]	UK	Retrospective cohort	ESUS	323 †	54.7 ± 17.4	61.0	Median 3	Mean 731 days	Multiple thresholds	Mean 731 days
Kreimer et al., 2024 [30]	Germany	Retrospective cohort	ESUS	101	58.9 ± 10.7	60.4	NR	Mean 647 days	≥30 s	Mean 647 days

† ESUS subgroup included in the analysis.

**Table 3:** Clinical Outcomes of the Included Studies.

Study	AF Detection Rate	Time to First AF Detection	Recurrent Stroke/TIA	Oral Anticoagulation	Device-Related Adverse Events
Randomized Controlled Trials					
Bernstein et al., 2021 [18]	15.7% (ICM) vs. 5.6% (control)	Majority detected within 6 months	Recurrent ischemic/hemorrhagic stroke and TIA reported at 12 months	Increased following AF detection	Implant-site infection, hemorrhage, and pain
Thijs et al., 2016 [19]	Progressive increase throughout 36-month follow-up	Most AF diagnosed within first 12 months	NR	Higher anticoagulant use after AF detection	No major device-related adverse events reported
Observational Studies					
Cotter et al., 2013 [21]	25.5%	Median 48 days (IQR 34–118)	NR	100% of AF patients switched to anticoagulation	None reported
Ziegler et al., 2017 [22]	4.6% (30 days); 12.2% (6 months); 16.3% (12 months); 21.5% (24 months)	Median 112 days (IQR 35–293)	NR	NR	NR
De Angelis et al., 2020 [23]	41.0% at 30 months	Mean 6 months after ICM implantation	One recurrent stroke (3%) in non-AF group; none in AF group	100% of AF patients anticoagulated	None reported
Öner et al., 2020 [24]	21.6%	Median 7 months (range 1–32)	5 patients (5.4%)	84% initiated oral anticoagulation	None specifically reported
Pecha et al., 2020 [25]	25.0% AF (35% overall arrhythmias)	NR	Four recurrent TIA/stroke events	All AF patients anticoagulated	Two device infections requiring explantation; one death during follow-up
Noubiap et al., 2023 [27]	5.5% (12 months); 8.9% (24 months); 14.0% (36 months)	Most AF detected >6 months after implantation	NR	NR	NR
Dulai et al., 2023 [26]	13.4% overall; 4.8% within 30 days	Mean 122.3 ± 121.7 days	NR	100% anticoagulated after AF detection	Five complications (2.7%), including one device explantation
Toyoda et al., 2024 [28]	1.5% (1 month); 6.0% (3 months); 13.6% (12 months); 18.0% (18 months); 28.2% (36 months)	Median 7.9 months (IQR 2.0–15.8)	11 patients (4.1%) developed recurrent stroke/TIA	60.5% initiated anticoagulation after AF detection	One battery depletion; no unexpected device-related complications
Chousou et al., 2024 [29]	48.6% (any AF); 32.2% (≥30 s); 14.9% (≥6 min); 6.8% (≥5.5 h)	Median 180 days (IQR 52–464)	NR	NR	No major adverse events reported
Kreimer et al., 2024 [30]	26.0%	Mean 231 ± 196 days	10 patients (9.9%); two had documented AF	100% anticoagulated following AF diagnosis	No device-related complications; five patients lost to follow-up

Abbreviations: AF, atrial fibrillation; ICM, implantable cardiac monitor; IQR, interquartile range; NR, not reported; TIA, transient ischemic attack.

**Table 4:** GRADE Summary of Findings.

Outcome	Studies (n)	Participants	Certainty of Evidence	Reasons for Downgrading
AF detection during ICM follow-up	10 observational	3651	Low **(⊕⊕◯◯)**	Observational design; very serious inconsistency (I^2^ = 95%)
Device-related adverse events	3 observational	351	Low **(⊕⊕◯◯)**	Observational design; imprecision due to few events and small sample size
Recurrent ischemic stroke/TIA	4 observational	524	Low **(⊕⊕◯◯)**	Observational design; imprecision from limited studies and events
Oral anticoagulation initiation after AF detection	7 observational	199 AF-positive patients	Very Low **(⊕◯◯◯)**	Observational design; serious inconsistency (I^2^ = 89.9%); imprecision

## Data Availability

The corresponding author can provide the supporting data for this study upon reasonable request.
